# The relationship between physical exercise and sense of social fairness among college students: the chain-mediated role of perceived social support and life satisfaction

**DOI:** 10.3389/fpsyg.2024.1430492

**Published:** 2024-08-20

**Authors:** Xiaodong Sui, Bin Zhao, Di Na, Jianwu Liu, Qi Zhang

**Affiliations:** ^1^School of Physical Education, Kunming University of Science and Technology, Kunming, China; ^2^School of Art, Yunnan Minzu University, Kunming, China; ^3^School of Physical Education, Hunan University of Technology, Zhuzhou, China; ^4^Oxbridge College, Kunming University of Science and Technology, Kunming, China

**Keywords:** physical exercise, perceived social support, life satisfaction, sense of social fairness, college students

## Abstract

**Background:**

The development of a stable society is closely linked to a prevalent sense of social fairness. Participating in physical activities, which are inherently social, plays a crucial role in fostering mental stability within social contexts.

**Objective:**

This study aims to examine how physical exercise influences the sense of social fairness among college students, focusing on the potential mediating effects of perceived social support and life satisfaction.

**Methods:**

The study surveyed 496 Chinese college students using several scales: the Physical Activity Rating Scale-3 (PARS-3), Perceived Social Support Scale (PSSS), Satisfaction with Life Scale (SWLS), and Social Justice Scale (SJS).

**Results:**

(1) A positive correlation was found between physical exercise and sense of social fairness (*r* = 0.151, *p* < 0.01). A significant direct effect of physical exercise on sense of social fairness was also observed (*β* = 0.151, *t* = 3.971, *p* < 0.01). (2) Physical exercise was a positive predictor of perceived social support (*β* = 0.113, *t* = 4.062, *p* < 0.01), which in turn positively influenced both life satisfaction (*β* = 0.333, *t* = 18.047, *p* < 0.01) and sense of social fairness (*β* = 0.485, *t* = 6.931, *p* < 0.01). Additionally, life satisfaction had a positive effect on sense of social fairness (*β* = 0.431, *t* = 3.247, *p* < 0.01). (3) Both perceived social support and life satisfaction significantly mediated the relationship between physical exercise and sense of social fairness through two pathways: physical exercise → perceived social support → sense of social fairness (mediating effect: 0.055); and physical exercise → perceived social support → life satisfaction → sense of social fairness (mediating effect: 0.016).

**Conclusion:**

(1) Physical exercise enhances both perceived social support and the sense of social fairness among college students, suggesting that it not only directly contributes to an enhanced sense of social fairness but also fosters supportive social relationships. (2) The influence of physical exercise on the sense of social fairness operates both directly and indirectly through the mediating roles of perceived social support and, sequentially, life satisfaction.

## 1 Introduction

Social stability is integral to a nation’s destiny and the wellbeing of its people. One of the primary strategies to mitigate social conflicts and foster stable social development is enhancing the citizens sense of social fairness. Physical exercise, as a form of social activity, plays a significant social role (Han and Huang, 2019; Huang et al., 2019) and may contribute positively to enhancing residents’ sense of social fairness.

A sense of fairness is crucial in guiding national governance and societal progress. Reflecting on the market reforms centered on economic development and efficiency prioritization, these policies have significantly fueled China’s rapid economic ascent. However, they have also led to increased social issues such as inequity, the widening wealth gap between the affluent and the poor, and growing disparities between urban and rural areas (Meng, 2012). These reforms have radically transformed the allocation of resources, forming the foundational basis for sense of fairness (Xu et al., 2020). Disparities in social fairness may precipitate social tensions and disrupt societal harmony and stability (Tao et al., 2015). Persistent unfairness can evoke intense negative emotions in individuals, indirectly heightening the perceived risk associated with unpredictable outcomes (Yang et al., 2017), thereby adversely impacting their physical and mental health, potentially leading to chronic illnesses, obesity, and depression (Hunte and Williams, 2009; Meier et al., 2009; Albert et al., 2010). Research has demonstrated an inverse relationship between social fairness and depression (Peker et al., 2018). Moreover, a robust sense of social fairness is instrumental in delaying and transforming the progression of depression, with a strong fairness perception mitigating the exacerbation of depressive symptoms and facilitating recovery (Wei et al., 2023). Su et al. (2019) discovered that high fairness perceptions are linked with positive emotional states, which help sustain a positive outlook and lessen depressive symptoms.

The influence of physical exercise on college students’ social mindfulness has attracted considerable scholarly attention, specifically regarding its impacts on subjective wellbeing (Tan et al., 2020), negative emotions (Liu, 2020), and life satisfaction (Stubbe et al., 2007). Research indicates that physical exercise not only enhances physical and mental wellbeing but also facilitates emotional exchange, reduces antagonism, and helps resolve conflicts, thereby stabilizing social attitudes (Zou and Sun, 2011). From the perspective of social capital theory, physical exercise also serves as a communal platform that fosters interaction, bolsters collective consciousness among residents, and helps mitigate negative social attitudes (Lei, 2020). Despite these findings, the specific effects of physical exercise on college students’ sense of social fairness—a key element in sustaining social stability—remain underexplored (Han and Huang, 2019). This gap highlights the uncertainty regarding how physical exercise influences sense of social fairness.

Current research into the mediating factors that influence the relationship between physical exercise and a sense of social fairness has focused on variables such as physical health and social capital (Sui et al., 2023). The findings indicate that these mediating variables exert only a partial influence, suggesting the existence of additional mediators that could impact this relationship. While previous studies have concentrated on objective factors as mediators influencing the connection between physical exercise and the perception of social fairness, there has been a lack of focus on subjective individual factors, especially among college student populations. Consequently, this study aims to examine the mediating roles of perceived social support and life satisfaction in how physical exercise influences college students’ sense of social fairness.

In conclusion, enhancing the sense of social fairness among college students is crucial not only for their mental health and social integration but also for the harmonious and stable progression of society. It is vital to delve into the underlying mechanisms of this relationship from both theoretical and empirical standpoints. This study aims to investigate the mediating mechanisms influencing the connection between physical exercise and students’ sense of social fairness.

### 1.1 Physical exercise and sense of social fairness

The notion of a sense of social fairness represents an individual’s basic perception or value judgment concerning the state of social fairness (Karni and Safra, 2002). This sense encompasses shifts in personal values and individual mentalities, as well as a psychosocial reaction to structural realities (Wegener, 1991). Scholars typically classify the sense of social fairness using two primary criteria: nature and connotation. The nature of social fairness can be segmented into fairness regarding power, opportunity, procedures, outcomes, and interactions (Cui, 2016), while the connotations of fairness are categorized into economic, political, educational, and social security aspects (Huang and Cui, 2018). Generally, Chinese residents exhibit a robust sense of social fairness (Xu et al., 2020); however, notable disparities exist among different societal groups. Research indicates that contemporary college students often display a relatively low sense of social fairness, influenced by variables such as gender, academic level, and familial background (Lv and Liu, 2009; Liu, 2021).

Physical exercise is recognized not only as a way to enhance physical capabilities but also to improve mental and emotional wellbeing. Its significance in fostering social fairness has been substantiated by various studies, highlighting its integral role in this domain (Darnell and Millington, 2019; Jarvie and Ahrens, 2019; Adamson et al., 2022). Theoretically, physical exercise supports the stabilization of social attitudes. It has been documented to positively affect college students’ interpersonal trust, self-efficacy, and psychological resilience, thereby decreasing the likelihood of experiencing negative emotions such as depression, anxiety, and stress (Qin and Dong, 2017; Liu, 2020). Moreover, physical exercise is credited with fostering social attitudes like wellbeing (Yasunaga et al., 2021), trust (Zhang et al., 2022), and life satisfaction (Yao et al., 2023). Furthermore, engaging in physical exercise plays a pivotal role in the creation and sustenance of social capital. Participants often develop a network of social relationships through these activities, which in turn enhances their social capital (Inman et al., 2015). Research by Sui et al. (2023) demonstrates that physical exercise can elevate the sense of social fairness by improving physical health and bolstering social capital. Based on this analysis, the study proposes Hypothesis 1: There is a positive correlation between physical exercise and the sense of social fairness among college students.

### 1.2 The mediating role of perceived social support

Perceived social support refers to an individual’s subjective perception and assessment of the extent of external support available to them, encompassing the emotional and perceptual experiences of feeling supported, understood, and respected (Zimet et al., 1988). Recognized as a valuable psychological resource, perceived social support not only mitigates job stress and burnout (Wei et al., 2016) but also aids in regulating daily negative emotions (Pottie and Ingram, 2008; Fang et al., 2021). According to self-determination theory, the social environment can boost individual motivation and its conversion by supporting the fulfillment of three fundamental psychological needs: autonomy, relationships, and competence. Past studies have identified perceived social support as a protective mediator that contributes to the development of a sense of fairness (Guo et al., 2021).

Given its inherently social nature and role in interpersonal interactions, physical exercise is a crucial means of enhancing social support (Lu, 2001). Numerous studies have established a significant positive correlation between college students’ physical exercise and their level of social support, demonstrating that physical exercise can enhance the social support experienced by college students (Wu et al., 2008; Chen et al., 2013). Participation in physical exercise influences college students’ daily activity patterns, increases their interaction frequency, affects their social network ties, and helps improve their social support networks. This, in turn, allows them to access greater social and emotional resources (Wang and Bo, 2023).

Perceived social support is known to significantly mitigate negative psychological traits in individuals (Fang et al., 2021). The buffering model of social support posits that social support can effectively cushion individuals against the impact of adverse conditions, offering a degree of protection from potential negative effects (Gnilka et al., 2019). Furthermore, the main effects model of social support asserts that consistent social support can confer mental health benefits, such as reducing symptoms of depression and enhancing overall wellbeing (Lakey and Orehek, 2011; Rueger et al., 2016; Ge et al., 2023). Based on these considerations, this study proposes Hypothesis 2: Perceived social support possibly acts as a mediating variable between physical exercise and the sense of social fairness among college students.

### 1.3 The mediating role of life satisfaction

Life satisfaction is defined as a stable, generalized cognitive appraisal of one’s overall life status or significant life aspects (Huebner, 2004), and it serves as a crucial indicator of an individual’s quality of life and subjective wellbeing (Wang and Zhang, 2012). Given their role as future societal leaders, the life satisfaction of college students has garnered interest from researchers across various disciplines including psychology, sociology, and economics (Liu et al., 2017; Peng et al., 2023). Previous findings indicate that life satisfaction among college students not only influences their emotional and behavioral issues but also has implications for future societal development (Zhou et al., 2020). Furthermore, life satisfaction is deeply intertwined with macro-environmental factors such as the quality of the social environment and economic conditions (Easterlin et al., 2012). Earlier studies have demonstrated that individuals who perceive fairness in the services they receive tend to report higher levels of satisfaction (Zhang, 2007).

Research increasingly focuses on the relationship between physical exercise and life satisfaction, particularly among students, where a positive association is consistently reported (Zullig and White, 2011; Maher et al., 2014; Pengpid and Peltzer, 2019). Studies have confirmed that regular physical exercise enhances life satisfaction in middle school students and long-distance runners (Gül and Küçükibis, 2018; Sato et al., 2018). However, in older adults, the impact of exercise on life satisfaction appears minimal, with friend support and self-efficacy emerging as significant factors (Dai and Yao, 2012). Additionally, lower life satisfaction is linked to negative coping styles and increased negative emotions (Proctor et al., 2009). A longitudinal study of individuals aged 18 to 35 identified life satisfaction as a predictor of mental health issues, including depression and substance dependence (Fergusson et al., 2015). The role of public services, such as healthcare and social security, is crucial as they contribute to social fairness, influencing life satisfaction through intergenerational, horizontal, and regional effects (Chen et al., 2022). This study proposes hypothesis 3 that life satisfaction might serve as a mediating factor between physical exercise and the sense of social fairness among college students.

### 1.4 The chain-mediating effect of perceived social support and life satisfaction

The main effect theory of social support suggests that an individual’s engagement with social resources significantly impacts their sense of purpose, belonging, and self-worth (Malecki and Demary, 2002). High levels of social support are seen as essential resources that foster individual development (Chen et al., 2018) and are linked to greater life satisfaction (Yang and Ye, 2014). Furthermore, the theory of embodied cognition posits that physical activities promote interactions that trigger specific mental states through sensory and motor system pathways (Yang et al., 2009). Research also indicates that individuals with robust social support perceive their organizations as just (Deconinck, 2010), and a strong belief in a just world correlates with higher life satisfaction (Kamble and Dalbert, 2012). Based on these insights, this study introduces Hypothesis 4: Perceived social support and life satisfaction sequentially mediate the relationship between physical exercise and the sense of social fairness among college students.

**Figure 1:**
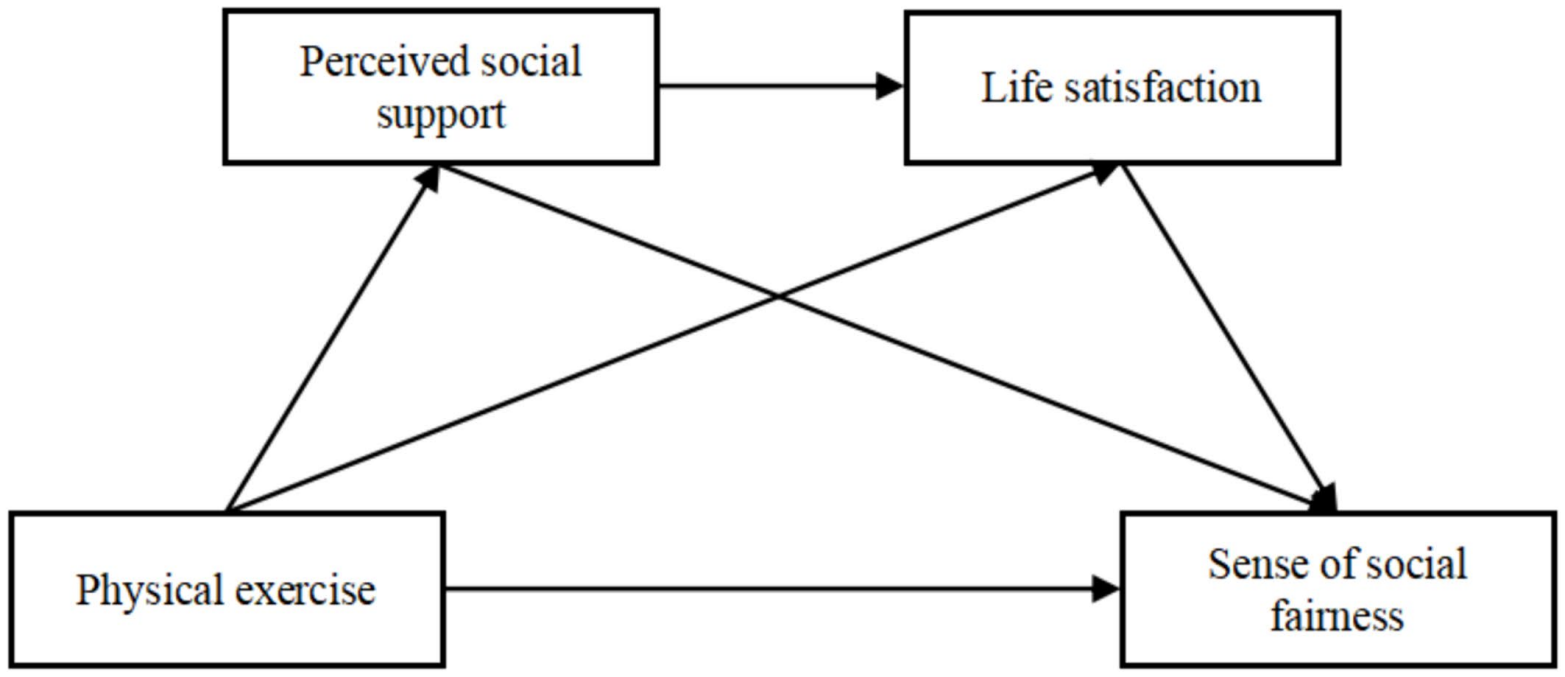
The hypothetical structure model.

This research framework, as shown in Figure 1, aims to: (1) assess the relationship between physical exercise and sense of social fairness among college students; (2) explore the role of perceived social support as a mediator between physical exercise and sense of social fairness; (3) investigate how life satisfaction mediates between physical exercise and sense of social fairness; and (4) examine the potential combined mediating effects of perceived social support and life satisfaction on this relationship.

## 2 Methods

### 2.1 Participants

Convenient sampling, being straightforward to implement, cost-effective, and fitting for exploratory research purposes in initial assessments, was employed in an offline questionnaire survey conducted at Kunming University of Science and Technology and Yunnan Minzu University. Participants voluntarily engaged in the survey, and subsequent to obtaining their informed consent, they were guided by trained personnel in a standardized manner to complete the questionnaires. After eliminating questionnaires with patterned responses and missing data, 496 out of the 550 distributed questionnaires were deemed valid, translating to an effective response rate of 90.18%. The exclusion criteria were: (1) incomplete questionnaires, and (2) responses containing logical inconsistencies. Based on these criteria, a sample fulfilling the survey prerequisites was established. The participants’ age ranged from 18 to 25 years, with a mean age of 20.50 years (SD = 1.58). The gender breakdown of the sample was 47.18% female and 52.82% male. In terms of academic standing, the sample comprised 146 freshmen (29.44%), 150 sophomores (30.24%), 127 juniors (25.60%), and 73 seniors (14.72%).

### 2.2 Measures

#### 2.2.1 Physical exercise

Physical exercise was assessed using the Physical Activity Rating Scale-3 (PARS-3) developed by Liang (1994). This scale evaluates three key aspects of exercise: intensity, duration, and frequency. Each aspect is captured by a single item, such as “How intense is your physical exercise?” Responses to each item are rated on a 5-point Likert scale. To compute the total score for physical exercise, the following formula is applied: Physical Exercise Level = Intensity of Exercise × (Duration of Exercise−1) × Frequency of Exercise. The possible scores on this scale range from 0 to 100, where higher scores reflect a greater level of physical exercise. The reliability of this scale in the current study was confirmed with a Cronbach’s alpha of 0.712.

#### 2.2.2 Perceived social support

The Perceived Social Support Scale (PSSS) devised by Zimet et al. (1988) was utilized to assess levels of perceived social support. This scale encompasses three facets of support: support from family, friends, and teachers. Each category is represented by four items, such as “I can get emotional help and support from my family when needed.” Responses are gauged on a 7-point Likert scale ranging from “strongly disagree” to “strongly agree.” Scores on the PSSS can vary from 0 to 84, where higher scores denote greater perceived social support. For the purposes of this study, the scale was administered in its Chinese-translated form, which has been previously validated for use in Chinese populations and demonstrated robust reliability and validity (Zhao and Li, 2017). The reliability of the scale in this investigation was high, with a Cronbach’s alpha of 0.929.

#### 2.2.3 Life satisfaction

Life satisfaction was assessed using the Satisfaction with Life Scale (SWLS) developed by Pavot and Diener (1993). This instrument consists of five items, such as “For the most part, my life is close to ideal.” Respondents rate each item on a 7-point Likert scale ranging from “strongly disagree” to “strongly agree.” The total scores on the SWLS can vary from 0 to 35, with higher scores reflecting greater life satisfaction. For this study, the SWLS was administered in its Chinese-translated version, which has been previously validated within Chinese populations, showing strong reliability and validity (Xiong and Xu, 2009). The reliability of the scale in the current study was confirmed with a Cronbach’s alpha of 0.873.

#### 2.2.4 Sense of social fairness

The Social Justice Scale (SJS), developed by Fang and Chen (2016), was employed to assess the sense of social fairness. This scale is structured into six dimensions: interactive fairness, procedural fairness, status fairness, distributive fairness, legal fairness, and opportunity fairness. Each dimension is represented by four items, such as “Individual income and social status are earned through personal endeavor and ability.” Respondents rated each item on a 5-point Likert scale, which ranges from “strongly disagree” to “strongly agree.” The total possible scores on the SJS can range from 0 to 120, with higher scores reflecting a stronger sense of social fairness. The reliability of the scale in this research was high, evidenced by a Cronbach’s alpha of 0.938.

### 2.3 Data analysis

Data analysis was conducted using SPSS 25.0 along with the SPSS macro-PROCESS. Initially, Harman’s single-factor analysis was performed to assess common method bias. Following this, descriptive statistics were generated, and the reliability of the scales was determined using Cronbach’s Alpha coefficient. Pearson correlation coefficients were then calculated to explore the relationships between the variables. To conclude the analysis, PROCESS (model 6) was utilized to investigate the chain mediation relationships among perceived social support, life satisfaction, physical exercise, and sense of social fairness.

## 3 Results

### 3.1 Common method bias tests

In this study, Harman’s single-factor test was utilized to assess the presence of common method bias, following the method outlined by Podsakoff et al. (2003). The analysis identified nine factors that each had eigenvalues exceeding 1. The first factor accounted for 33.91% of the total variance, which is below the often-used critical threshold of 40%. Based on these findings, it can be concluded that common method bias does not pose a significant concern in this research.

### 3.2 Descriptive statistics and correlations

Table 1 presents the mean values, standard deviations, and correlation coefficients for physical exercise, perceived social support, life satisfaction, and sense of social fairness. The results indicate significant correlations among all variables. Specifically, physical exercise is weakly and positively associated with perceived social support, life satisfaction, and sense of social fairness. Both perceived social support and life satisfaction demonstrate strong positive correlations with sense of social fairness. Additionally, there is also a strong positive correlation between perceived social support and life satisfaction.

**Table 1:** Descriptive statistics and correlations.

Variable	*M*	*SD*	1	2	3	4
1. Physical exercise	20.292	18.502	1			
2. Perceived social support	63.873	11.403	0.179***	1		
3. Life satisfaction	22.960	5.891	0.139***	0.636***	1	
4. Sense of social fairness	92.851	15.696	0.166***	0.495***	0.399***	1

*N* = 496. ***p < 0.01; all tests were two-tailed.

### 3.3 The chain mediating effects analysis

The data was found to be normal as all of the absolute values of the skewness (range: −0.508 to 1.522) and kurtosis (range: −1.955 to 2.560) indices were less than 2 and 7, respectively (Kline, 2023). The PROCESS v4.1 macro, Model 6 by Hayes (2012), was utilized to examine the mediating effects, using perceived social support and life satisfaction as mediators, physical exercise as the independent variable, and sense of social fairness as the dependent variable. Gender, age, and grade were included as control variables using a stepwise regression method. The results are detailed in Table 2, the variance inflation factors of all independent variables in the model are conclusively under the threshold of 5, thereby indicating the absence of any issue pertaining to multicollinearity.

**Table 2:** Regression analysis of the relationship between physical exercise and sense of social fairness.

Outcome	Predictor	*R^2^*	*F*	*β*	*t*	95% Boot CI
Sense of social fairness	Gender	0.078	10.330^***^	−4.412	−3.097^***^	[−7.212, −1.613]
	Age			0.917	1.163	[−0.631, 2.465]
	Grade			−3.383	−2.813^***^	[−5.746, −1.020]
	Physical exercise			0.151	3.971^***^	[0.076, 0.225]
Perceived social support	Gender	0.062	8.050^***^	−2.047	−1.961^*^	[−4.0.099, 0.004]
	Age			1.138	1.971^**^	[0.003, 2.273]
	Grade			−2.641	−2.996^***^	[−4.373, −0.909]
	Physical exercise			0.113	4.062^***^	[0.058, 0.167]
Life satisfaction	Gender	0.413	68.892^***^	0.841	1.962^*^	[−0.001, 1.684]
	Age			−0.077	−0.324	[−0.543, 0.389]
	Grade			0.313	0.861	[−0.402, 1.028]
	Physical exercise			0.004	0.387	[−0.018, 0.027]
	Perceived social support			0.333	18.047^***^	[0.297, 0.369]
Sense of social fairness	Gender	0.288	33.036^***^	−3.489	−2.761^***^	[−5.972, −1.006]
	Age			0.235	0.337	[−1.133, 1.603]
	Grade			−1.859	−1.739^*^	[−3.960, 0.241]
	Physical exercise			0.078	2.291^**^	[0.011, 0.144]
	Perceived social support			0.485	6.931^***^	[0.347, 0.622]
	Life satisfaction			0.431	3.247^***^	[0.170, 0.691]

*N* = 496. Boot confidence interval (CI) upper limit (ULCI), and boot CI lower limit (LLCI) are indicative of the standard deviation (SD) of the indirect effect evaluated through the deviation correction percentile Bootstrap approach and the upper and lower limits of the respective 95% confidence intervals (CIs). CI, confidence interval. *p < 0.1; **p < 0.05; ***p < 0.01.

According to Table 2, physical exercise has a weak positive relationship with college students’ sense of social fairness (*β* = 0.151, *p* < 0.01), supporting Hypothesis 1. Introducing perceived social support into the model shows that physical exercise weakly and positively predicts perceived social support (*β* = 0.113, *p* < 0.01), and in turn, perceived social support strongly and positively influences sense of social fairness (*β* = 0.485, *p* < 0.01), thus confirming Hypothesis 2. When life satisfaction is added to the model, it strongly and positively predicts sense of social fairness (*β* = 0.431, *p* < 0.01), although the direct effect of physical exercise on life satisfaction is not significant (*β* = 0.004, *p* > 0.1), leading to a rejection of Hypothesis 3. However, the addition of both mediators into the equation shows that perceived social support strongly and positively affects life satisfaction (*β* = 0.333, *p* < 0.01), suggesting a chain mediation and confirming Hypothesis 4.

**Figure 2:**
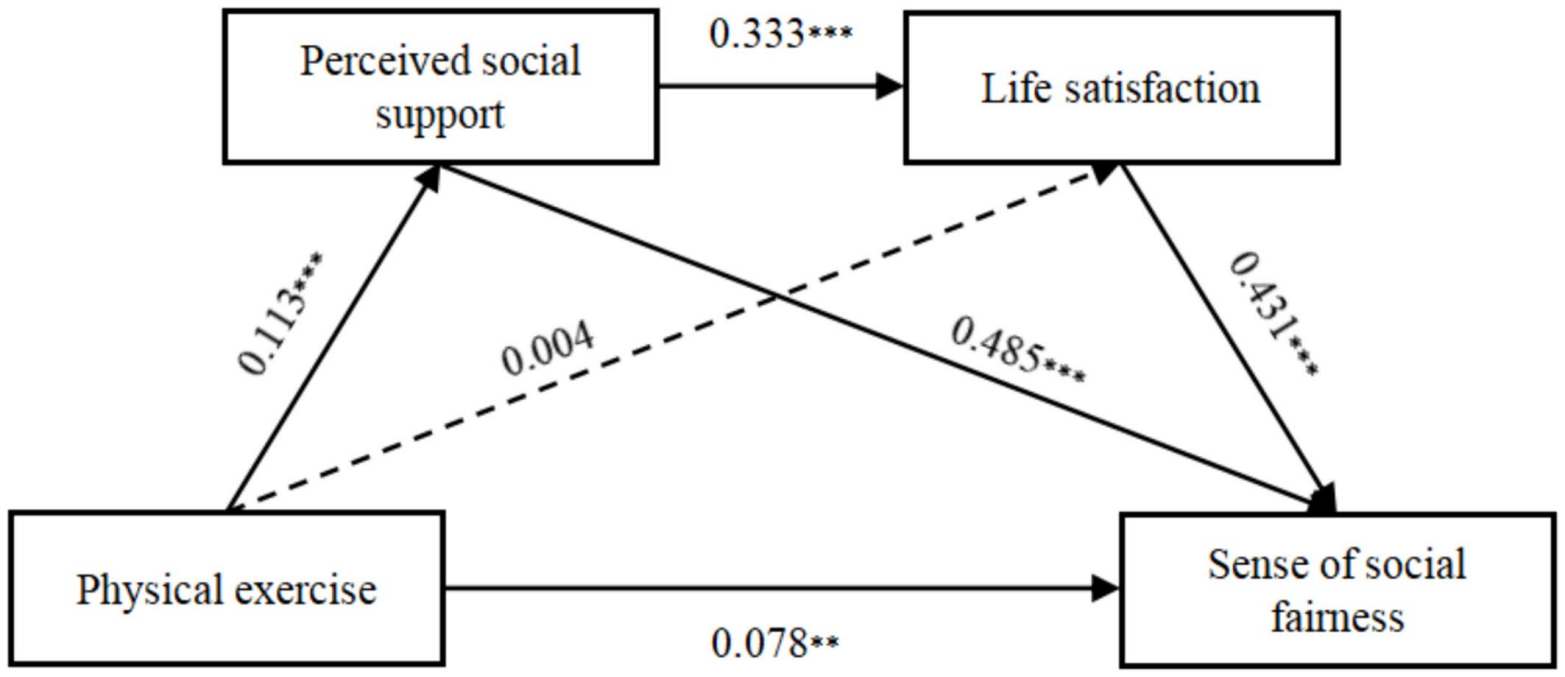
The chain mediation effect of perceived social support and life satisfaction. **p < 0.05; ***p < 0.01.

To further elucidate the mediation pathways, bias-corrected bootstrap tests with 5,000 samples were conducted, providing 95% confidence intervals. These intervals were all exclusive of zero for the mediating effects, indicating significant mediation. The total effect of physical exercise on sense of social fairness was 0.151, with a direct effect of 0.078. The total indirect effect accounted for 48.34% of the total effect, calculated as 0.073 (Table 3). This total mediation included three pathways: Pathway 1 (physical exercise → perceived social support → sense of social fairness) with an indirect effect of 0.055; Pathway 2 (physical exercise → life satisfaction → sense of social fairness) with an indirect effect of 0.002 (insignificant, as its 95% confidence interval includes zero); and Pathway 3 (physical exercise → perceived social support → life satisfaction → sense of social fairness) with an indirect effect of 0.016. The significant pathways further validate Hypotheses 2 and 4. These mediation effects are graphically represented in Figure 2.

**Table 3:** Perceived social support and life satisfaction in the mediation effect analysis.

	Indirect effects	Boot SE	Boot LLCI	Boot ULCI	Relative mediation effect
Total indirect effect	0.073	0.019	0.036	0.111	48.34%
Indirect effect 1	0.055	0.160	0.026	0.088	36.42%
Indirect effect 2	0.002	0.006	−0.009	0.014	
Indirect effect 3	0.016	0.007	0.005	0.030	10.60%
Compare 1	0.053	0.017	0.021	0.088	
Compare 2	0.039	0.016	0.010	0.074	
Compare 3	−0.014	0.009	−0.034	−0.001	

*N* = 496. Boot SE, Boot LLCI, and Boot ULCI refer, respectively, to the standard error and the lower and upper limits of the 95% confidence interval of the indirect effects estimated by the percentile bootstrap method with deviation correction.

## 4 Discussion

### 4.1 The relationship between physical exercise and sense of social fairness

This study confirmed that physical exercise is significantly and positively associated with college students’ sense of social fairness with an effect value of 0.078, thus supporting Hypothesis 1. This finding aligns with previous research (Lee and Cunningham, 2019; Adamson et al., 2022), which also observed a positive influence of physical exercise on students’ sense of social fairness. Sui et al. (2023) found an effect value of 0.069 between physical exercise and residents’ sense of social fairness. This suggests a stronger relationship between physical exercise and college students’ sense of social fairness. It has been demonstrated that physical exercise not only enhances physical health but also contributes to mental and social stability (Sui et al., 2023). Firstly, engaging in physical exercise can reduce the risk of depression and promote health equity, which are crucial for maintaining optimal physical functioning and enhancing sense of social fairness (Levy and Patz, 2015; Fang and Guo, 2019). Secondly, physical exercise positively affects the development of trust, a fundamental component of social capital and a significant factor influencing sense of social fairness (Seippel, 2006; Inman et al., 2015). These factors illustrate how physical exercise can significantly boost the sense of social fairness among college students. Therefore, encouraging active participation in physical exercise not only benefits students’ physical health but also fosters a healthier social mentality and promotes stable social development.

### 4.2 The mediating effect of perceived social support

This study confirmed that perceived social support serves as an indirect link between physical exercise and college students’ sense of social fairness, validating Hypothesis 2. Consistent with prior research (Wu et al., 2008; Chen et al., 2013), this study found that physical exercise significantly boosts the level of perceived social support among college students. Further, literature suggests that enhanced perceived social support can foster a positive mentality in college students (Ratelle et al., 2013; Chen and Wu, 2015), a finding echoed in research involving college students with disabilities (He et al., 2021). The findings of this study align with the social interaction hypothesis regarding the psychological effects of physical exercise. Specifically, engaging in physical exercise facilitates positive interpersonal interactions and fosters the generation of positive emotions. The development or enhancement of social network structures through conceptual exchanges, interpersonal interactions, and emotional connections during physical exercise, along with the support derived from these activities, positively affects individuals’ relational skills, emotional regulation, and stress management capabilities (Cohen et al., 2000). These positive interpersonal relationships boost college students’ social capital and perceived social support, subsequently enhancing their sense of social fairness (Wang, 2020). Thus, it is evident that physical exercise can significantly elevate college students’ sense of social fairness through the increased levels of perceived social support. This underscores the importance of encouraging physical exercise among college students not only for physical health benefits but also for its positive impacts on social and psychological wellbeing.

### 4.3 The mediating effect of life satisfaction

This study found no evidence to support the mediating role of life satisfaction in the relationship between physical exercise and sense of social fairness, leading to the rejection of Hypothesis 3. The influence of physical exercise on life satisfaction appears to be a complex process that is not direct but mediated through various factors related to physical, psychological, and social functioning (Elavsky et al., 2005; Mcauley et al., 2006; Motl et al., 2009; Motl and Mcauley, 2009). According to Li et al. (2022), while extracurricular physical exercise does not have a direct impact on life satisfaction, it does so indirectly by enhancing adolescents’ self-confidence and resilience. Further research into older populations underscores this complexity; a study found no direct link between physical exercise and life satisfaction, pointing instead to friend support and self-efficacy as critical mediating variables (Dai and Yao, 2012). Life satisfaction is fundamentally a cognitive evaluation of one’s life circumstances. The pathway from individual behavior to cognitive assessment typically involves multiple mediating factors. Therefore, it is plausible that during physical exercise, perceived changes in behavioral competence, value, and the individual’s capacity to manage stress and trauma could act as complete mediators, shaping their overall life satisfaction judgments. This suggests that enhancing life satisfaction through physical exercise requires a broader understanding of the interplay between various psychological and social factors.

### 4.4 The chain mediation effect of perceived social support and life satisfaction

This study confirmed that perceived social support and life satisfaction mediate the relationship between physical exercise and college students’ sense of social fairness, validating Hypothesis 4. Essentially, physical exercise indirectly influences college students’ sense of social fairness through enhancements in their perceived social support and life satisfaction. According to the main and buffering effects theories of social support, social support not only positively influences the development of an individual’s mental health but also mitigates the negative impacts of stress on health (Cohen and Wills, 1985; Uchino, 2006). Furthermore, engagement in physical exercise bolsters college students’ sense of trust and social participation, thereby enhancing their social capital (Qin and Dong, 2017). Sui et al. (2023) found that social capital significantly promotes participation in physical exercise and enhances sense of social fairness. Additionally, the sense of social fairness has been shown to be closely linked with subjective emotional states, where higher levels of positive emotions and life satisfaction are associated with stronger sense of social fairness (Jian et al., 2020; Martion and Prilleltensky, 2020). The findings of this study underscore the intricate link between physical exercise and social fairness, highlighting how physical exercise can significantly impact college students’ sense of social fairness through the mediating roles of social support and life satisfaction. This suggests that interventions aimed at increasing physical exercise among college students could beneficially influence their mental health and societal perceptions, thereby fostering a more equitable social environment. In conclusion, the chain mediation of perceived social support and life satisfaction is pivotal in connecting physical exercise with college students’ sense of social fairness.

### 4.5 Practical significance

This study investigated the connection between physical exercise and the sense of social fairness among college students, examining the roles of perceived social support and life satisfaction as mediators. The findings demonstrate that physical exercise not only has a direct positive effect on sense of social fairness but also influences it indirectly through the mediating effects of perceived social support and a chain mediation involving both perceived social support and life satisfaction. This research highlights how physical exercise impacts college students’ sense of social fairness and outlines potential mechanisms, which is crucial for fostering a positive social mentality among this demographic.

The results underscore the importance of considering both the direct and indirect effects of physical exercise in enhancing college students’ sense of social fairness. To practically apply these findings, several strategies can be implemented: (1) Encourage active participation: colleges should promote and facilitate regular physical exercise by organizing sports events and providing appropriate exercise guidance. This not only helps in physical fitness but also boosts perceived social support, life satisfaction, and subsequently, the sense of social fairness among students. (2) Enhance social support: social support is an effective tool for fostering a positive mentality. It is important to create an environment where students feel supported; this can be achieved through mentoring programs, counseling services, and building strong community networks. Ensuring that students who perceive low social fairness can access and benefit from these supports is vital for improving their life satisfaction and sense of fairness.

Cultivating a positive social mentality has profound implications for individual growth and societal advancement: Improving self-confidence and adaptability: (1) Developing a positive social mentality helps students to better manage life’s challenges and adapt to changes, enhancing their overall resilience. (2) Boosting social responsibility: it encourages a heightened sense of community and civic duty among students, preparing them to contribute positively to societal development. (3) Promoting societal progress: by fostering a generation of mentally positive individuals, societies can enhance collective happiness and improve the quality of life for all members. This study’s insights into the interplay between physical exercise, perceived social support, life satisfaction, and social fairness offer valuable guidance for educational institutions aiming to nurture well-rounded individuals who are both physically active and socially conscious.

## 5 Limitations and prospects

The results of this study hold significant theoretical value and practical implications; however, there are several limitations that warrant mention. First, the cross-sectional design of this study limits the ability to establish causality. Although the relationships explored among variables are supported by prior research, establishing a causal linkage requires longitudinal or experimental designs that track changes over time. Second, in this study, convenience sampling was employed, which resulted in an arbitrary determination of the sample and did not adequately represent a well-defined population. Therefore, more rigorous and scientific sampling methods will be adopted in future research. Third, the measurement methods employed were relatively uniform. Future research could enhance the validity of results by incorporating more direct and varied measures of physical exercise, such as objectively monitoring the frequency, duration, intensity, and total amount of physical exercise, beyond self-reported data from the Physical Activity Rating Scale. Lastly, while this study focused on perceived social support and life satisfaction as mediators between physical exercise and sense of social fairness, other potential mediating variables exist. Factors such as self-esteem, self-efficacy, peer relationships, academic pressures, and physical health could also play significant roles in shaping students’ sense of social fairness. These factors should be explored in future studies to provide a more comprehensive understanding of the pathways through which physical exercise impacts social fairness among college students. Addressing these limitations in subsequent research will enhance the robustness of findings and help in developing more targeted interventions aimed at improving sense of social fairness through physical exercise and related psychosocial factors.

## 6 Conclusion

In conclusion, this study underscores the importance of physical exercise as a significant factor influencing college students’ sense of social fairness. It was found that the relationship between physical exercise and sense of social fairness among college students is potentially mediated by perceived social support and life satisfaction. These findings enrich our understanding of the mechanisms through which physical exercise can enhance sense of social fairness and suggest that engaging in physical exercise may be beneficial in fostering a more equitable mindset among students. Moreover, the mediating roles of perceived social support and life satisfaction highlight complex psychosocial processes that contribute to these effects, providing a foundation for further empirical studies. This research not only adds to the existing literature by demonstrating the positive effects of physical exercise on social fairness but also offers practical insights for designing interventions aimed at increasing the sense of fairness through enhanced social support and improved life satisfaction among college populations. Future studies should aim to address the limitations noted in the current research, such as its cross-sectional design, and explore additional mediating variables that may impact this relationship. Such investigations will help refine strategies for promoting social fairness and wellbeing through physical exercise, ultimately contributing to healthier, more socially responsive educational environments.

## Data Availability

The raw data supporting the conclusions of this article will be made available by the authors, without undue reservation.
